# Assessment of reproducibility and biological variability of fasting and postprandial plasma metabolite concentrations using ^1^H NMR spectroscopy

**DOI:** 10.1371/journal.pone.0218549

**Published:** 2019-06-20

**Authors:** Ruifang Li-Gao, David A. Hughes, Saskia le Cessie, Renée de Mutsert, Martin den Heijer, Frits R. Rosendaal, Ko Willems van Dijk, Nicholas J. Timpson, Dennis O. Mook-Kanamori

**Affiliations:** 1 Department of Clinical Epidemiology, Leiden University Medical Center, Leiden, the Netherlands; 2 MRC Integrative Epidemiology Unit, School of Social and Community Medicine, University of Bristol, Bristol, United Kingdom; 3 Department of Biomedical Data Sciences, Leiden University Medical Center, Leiden, the Netherlands; 4 Department of Internal Medicine, VU Medical Center, Amsterdam, The Netherlands; 5 Department of Internal Medicine, division of Endocrinology, Leiden University Medical Center, Leiden, the Netherlands; 6 Einthoven Laboratory for Experimental Vascular Medicine, Leiden University Medical Center, Leiden, the Netherlands; 7 Department of Human Genetics, Leiden University Medical Center, Leiden, the Netherlands; 8 Department of Public Health and Primary Care, Leiden University Medical Center, Leiden, the Netherlands; University of Illinois, UNITED STATES

## Abstract

**Introduction:**

It is crucial to understand the factors that introduce variability before applying metabolomics to clinical and biomarker research.

**Objectives:**

We quantified technical and biological variability of both fasting and postprandial metabolite concentrations measured using ^1^H NMR spectroscopy in plasma samples.

**Methods:**

In the Netherlands Epidemiology of Obesity study (n = 6,671), 148 metabolite concentrations (101 metabolites belonging to lipoprotein subclasses) were measured under fasting and postprandial states (150 minutes after a mixed liquid meal). Technical variability was evaluated among 265 fasting and 851 postprandial samples, with the identical blood plasma sample being measured twice by the same laboratory protocol. Biological reproducibility was assessed by measuring 165 individuals twice across time for evaluation of short- (<6 months) and long-term (>3 years) biological variability. Intra-class coefficients (ICCs) were used to assess variability. The ICCs of the fasting metabolites were compared with the postprandial metabolites using two-sided paired Wilcoxon test separately for short- and long-term measurements.

**Results:**

Both fasting and postprandial metabolite concentrations showed high technical reproducibility using ^1^H NMR spectroscopy (median ICC = 0.99). Postprandial metabolite concentrations revealed slightly higher ICC scores than fasting ones in short-term repeat measures (median ICC in postprandial and fasting metabolite concentrations 0.72 versus 0.67, Wilcoxon p-value = 8.0×10^−14^). Variability did not increase further in a long-term repeat measure, with median ICC in postprandial of 0.64 and in fasting metabolite concentrations 0.66.

**Conclusion:**

Technical reproducibility is excellent. Biological reproducibility of postprandial metabolite concentrations showed a less or equal variability than fasting metabolite concentrations over time.

## Introduction

Metabolomics, as one of the pillars within the “omics” technologies, have been widely investigated in the search for disease predictors in clinical and preclinical studies [1–3]. Despite growing interest, few findings from metabolomics studies have been translated into clinical prognosis and diagnosis routines. This may be at least partly due to concerns regarding the validity and reliability of the metabolomics measurements. Understanding the sources of metabolite level variations therefore is crucial for accelerating the translation from research to clinical applications [4].

The variability attributable to inter-individual variation is normally defined as reproducibility [5] and expressed by the intra-class correlation coefficient (ICC) [6]. The fluctuations of metabolite concentrations are known to be affected by diet, season, circadian rhythm, and menstrual cycle [7], which may mask the real physiological variations due to disease status or interventions. Nonetheless, previous studies reported relatively stable short-term metabolic profiles for both plasma and urine specimens across different metabolomics platforms [7–10]. Floegel et.al. assessed the reproducibility of 163 fasting plasma metabolite concentrations over a 4-month period and observed a median ICC of 0.57 [8]. Similarly, using a ^1^H Nuclear Magnetic Resonance spectroscopy (NMR)-based platform, Nicholson et.al. showed that nearly 60% of the biological variation could be attributed to stable familial and individual-environment factors in a twin study [9]. Another recent study on children’s urine samples analyzed six days apart, reported a median ICC of 0.40 across 44 ^1^H NMR-based metabolite concentrations [10]. Sampson et. al. investigated the sources of variability in both fasting and naturally non-fasting plasma metabolite measurements one year apart using liquid chromatography/mass spectrometry (LC/MS) and gas chromatography mass spectroscopy (GC/MS) platforms, and over 60% of variability was attributable to intra-individual and assay variability [7].

Cardiovascular risk prediction and other biomarker research is generally limited to samples taken in the fasting state. However, others and we have also begun to explore the utility of non-fasting measures [11, 12]. Non-fasting samples have the logistic advantage that the participant or patient does not have to fast over a prolonged period. Moreover, non-fasting measures may be more predictive for disease since the human body resides in a non-fasting state for the majority of the day, or both may individually be predictive. However, the within-individual and between-individual variability of postprandial metabolite concentrations is unknown.

In this study, we extend the previous metabolomics reproducibility studies by (1) taking both fasting and postprandial metabolite concentrations into account to evaluate variability and 2) considering both short- and long-term repeat measures (<6 months and >3 years, respectively) of reproducibility. In short, we aim to estimate how much technical and biological variability there is in metabolite concentrations when sampling an individual in fasting and postprandial states as well as when repeating the experiment in the same individual over two different time intervals.

## Materials and methods

### Study population

The study was embedded in a population-based prospective cohort, the Netherlands Epidemiology of Obesity (NEO) study [13]. All participants gave written informed consent and the Medical Ethical Committee of the Leiden University Medical Center (LUMC) approved the study design. Initiated in 2008, NEO was designed to study pathways that lead to obesity-related diseases. Detailed information about the study design and data collection has been described elsewhere [13]. In brief, men and women aged between 45 and 65 years with a self-reported body mass index (BMI) of 27 kg/m^2^ or higher living in the greater area of Leiden (in the west of the Netherlands) were eligible to participate in the NEO study. In addition, all inhabitants aged between 45 and 65 years from one municipality (Leiderdorp) were invited irrespective of their BMI. Participants were invited for a baseline visit at the NEO study center in the LUMC after an overnight fast. Prior to their visits, participants completed a questionnaire at home with demographic, lifestyle and clinical data. Fasting blood samples were drawn after an overnight fast. Within the next five minutes after the fasting blood draw, a liquid mixed meal (400mL, 600 kcal, with 16 percent of energy (En%) derived from protein, 50 En% carbohydrates, and 34 En% fat) was consumed and subsequent blood samples were drawn 30 and 150 minutes after the meal, processed within 4 hours and stored at -80 degrees Celsius.

### Technical and biological reproducibility validation subsample

Frozen serum extracts were shipped to the University of Bristol, UK, in freezer boxes containing 94 samples each. NMR-based metabolomics processing included a single thaw cycle, followed by lipid extractions performed by a single technician, and a subsequent sample preparation carried out by two technicians, with all liquid handling being performed on a Janus liquid handler. All samples of a single sample collection were processed all together. However, the order that the boxes were processed was randomised.

Two hundred and sixty-five fasting and 851 postprandial randomly selected technical replicates (duplicate serum aliquots prepared in Leiden) were processed at the University of Bristol, UK, in February of 2015 and again in October of 2016. These technical replicates provide a means to evaluate error that may be introduced by the NMR platform when repeating all NMR-processing steps on two aliquots of the same biological sample with a 20-month gap.

Biological or experimental repeats, that is repeating the entire experiment—food challenge and all—on a sub-sample of NEO participants, were also collected. From 2011 to 2012, 183 NEO participants were invited for a validation study of the baseline measures, and their blood samples were collected twice across time. Short-term biological reproducibility is specified as those resampled within 6 months, and long-term was determined as an interval longer than 3 years. For the current analyses, individuals were included based on their compliance to (1) overnight fasting (one individual excluded); (2) the meal challenge protocol (4 individuals excluded) and (3) two repeated measures over time after exclusions (13 individuals excluded). After selection, 87 short-term biological repeat and 78 long-term biological repeat participants were included in the analyses of biological repeatability. Since the validation project was conceived in the later phase of the NEO study, most of the revisits occurred in the last year of the NEO baseline collection, i.e. between 2011 and 2012.

### NMR spectroscopy-based plasma metabolite quantification

Metabolomic measurements were performed in both fasting and postprandial (t = 150 minutes after the liquid meal) plasma samples using the Nightingale high-throughput NMR metabolomics platform [14]. No metabolomic measurements at the 30-minute sampling interval were measured. The metabolomics platform provides 148 metabolites (S1 Table) from eleven substance classes: lipoprotein subclasses (n = 98), lipoprotein particle sizes (n = 3), apolipoproteins (n = 2), fatty acids and saturation (n = 11), cholesterol (n = 9), glycerides and phospholipids (n = 9), amino acids (n = 8), ketone bodies (n = 2), inflammation (n = 1), glycolysis related metabolites (n = 3), and fluid balance (n = 2). The NMR-based metabolomics platform and the experimental procedure have been described in details previously [15]. To remove samples with low blood quality and measurement errors, individuals were excluded when (1) metabolite concentrations deviated more than 4 standard deviation of the mean values derived from the entire NEO population and (2) more than 30% of missingness on all 148 metabolite concentrations under either fasting or postprandial states. To correct for batch effects (mainly due to ambient temperature and seasonal variability), blood sampling date was entered as a variable in a linear regression model on the metabolite concentrations in the entire NEO population (N = 6,350). The following analyses on metabolite concentrations were based on the residuals from that linear regression model. Residual metabolite concentrations were not further log-transformed as the mean estimated Shapiro-Wilk W-statistics was 0.97 and only 3 metabolites exhibited a value smaller than 0.90. In addition, we observed that the commonly used log-transformations made the distributions less normal with W-statistics smaller (mean W-statistic = 0.92) than untransformed data (Student’s t-test, p-value <0.001).

### Statistical analyses

**Fig 1:**
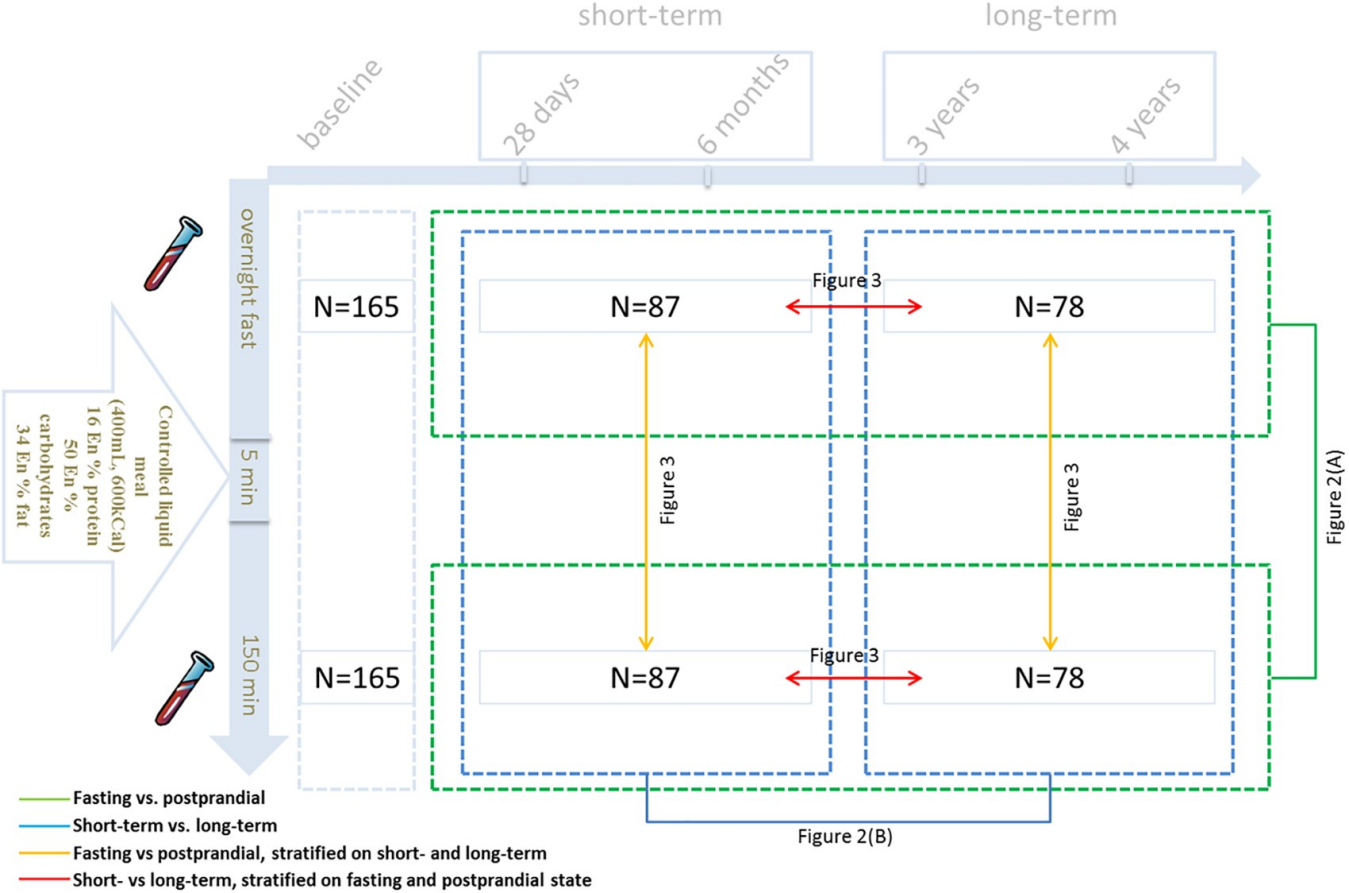
Workflow of study design and statistical analyses for biological replication.

For each metabolite, intra-class correlation coefficients (ICC) were calculated to evaluate technical and biological variability separately for fasting and postprandial samples. ICC as the measurement of variability is defined as the ratio of between-subject variance to the total variance composed of sum of between- and within-subject variance, which was calculated by one-way ANOVA random effect model. The corresponding 95% confidence intervals (CIs) were derived from bootstrapping using “ICCest” function in the R package “ICC” [6]. The reproducibility was classified based on the ICCs as follows: excellent > 0.75, good 0.51–0.74, fair 0.40–0.50, and poor <0.40 [8]. ICC scores were calculated for each fasting and postprandial metabolite separately (irrespective of short-/long-term time interval). The ICC score distributions composed of 149 metabolite concentrations were compared between fasting and postprandial states by two-sided paired Wilcoxon test. The p-value and nonparametric 95% confidence interval (CI) of location shifts were calculated by the medians of the differences of the resampled postprandial metabolites minus the resampled corresponding fasting metabolites with bootstrapping. Similarly, metabolite measurements’ variability over time was compared between short- and long-term repeated measures regardless of fasting/postprandial state. In addition, the interaction between fasting/postprandial state and short-/long-term time intervals was examined by comparisons of fasting and postprandial ICC distributions, stratified on short- and long-term respectively (Fig 1). To further decompose metabolite level variations, for each metabolite a linear mixed effect model was used to model the by-subject random slope for within-subject variations. The defined fixed effects were age, sex, the fasting/postprandial state and time intervals (short-/long-term). The linear mixed model was calculated by the function lmer in the R package “lme4” [16]. The proportion of variance explained by random effects was estimated by the function VarCorr in the R package “lme4”.

## Results

### Sample summaries

Summaries of baseline characteristics of all the participants in this reproducibility study are presented in Table 1. For individuals in the short-term biological reproducibility analysis, the median time between the first and second visit was 105 days, and 41% were men. In the long-term biological reproducibility analysis, the median time interval between two visits was 1,305 days and 67% were men. The median BMI did not change over the two visits (short-term: 26.6 kg/m^2^ vs. 26.1 kg/m^2^; long-term: 30.4 kg/m^2^ vs. 30.2 kg/m^2^).

**Table 1:** Characteristics of NEO reproducibility sub-population.

	Technical reproducibility*		Biological reproducibility			
	Fasting	Postprandial	Short-term (<6 months)		Long-term (>3 years)	
			1^st^ visit**	2^nd^ visit**	1^st^ visit**	2^nd^ visit**
N	265	851	87		78	
Age (years)	57 [51, 61]	56 [50, 61]	59 [54, 62]	59 [54, 62]	57 [52, 61]	60 [56, 64]
Sex (Men%)	147 (55.5%)	397 (46.7%)	36 (41.4%)		52 (66.7%)	
BMI (kg/m^2^)	30.5 [28.5, 33.3]	29.0 [26.7, 31.9]	26.6 [23.9, 28.9]	26.1 [23.7, 28.8]	30.4 [28.6, 32.8]	30.2 [28.6, 32.5]
Visit interval (days)	NA		105 [95, 119]		1,305 [1,282, 1,333]	

Continuous variables were represented by median (IQR).

* technical reproducibility used repeat fasting and postprandial blood samples from baseline.

** the individuals visited twice

### Technical reproducibility

In total 1,116 (265 fasting and 851 postprandial) samples were technically repeated, with the same blood plasma sample processed twice with the same laboratory protocols. With the exception of a single metabolite, we observed excellent technical reproducibility with an average ICC score above 0.99, in both fasting and postprandial states. As 101 out of 159 considered metabolites belong to the same category of lipoprotein subclasses and sizes and share high correlations among each other, the high average ICC score reflected the metabolite correlations accordingly. Only the metabolite related to estimated description of fatty acid chain length exhibited lower ICC values of 0.77 and 0.83 in fasting and postprandial states, respectively.

### Fasting/Postprandial state reproducibility

**Fig 2:**
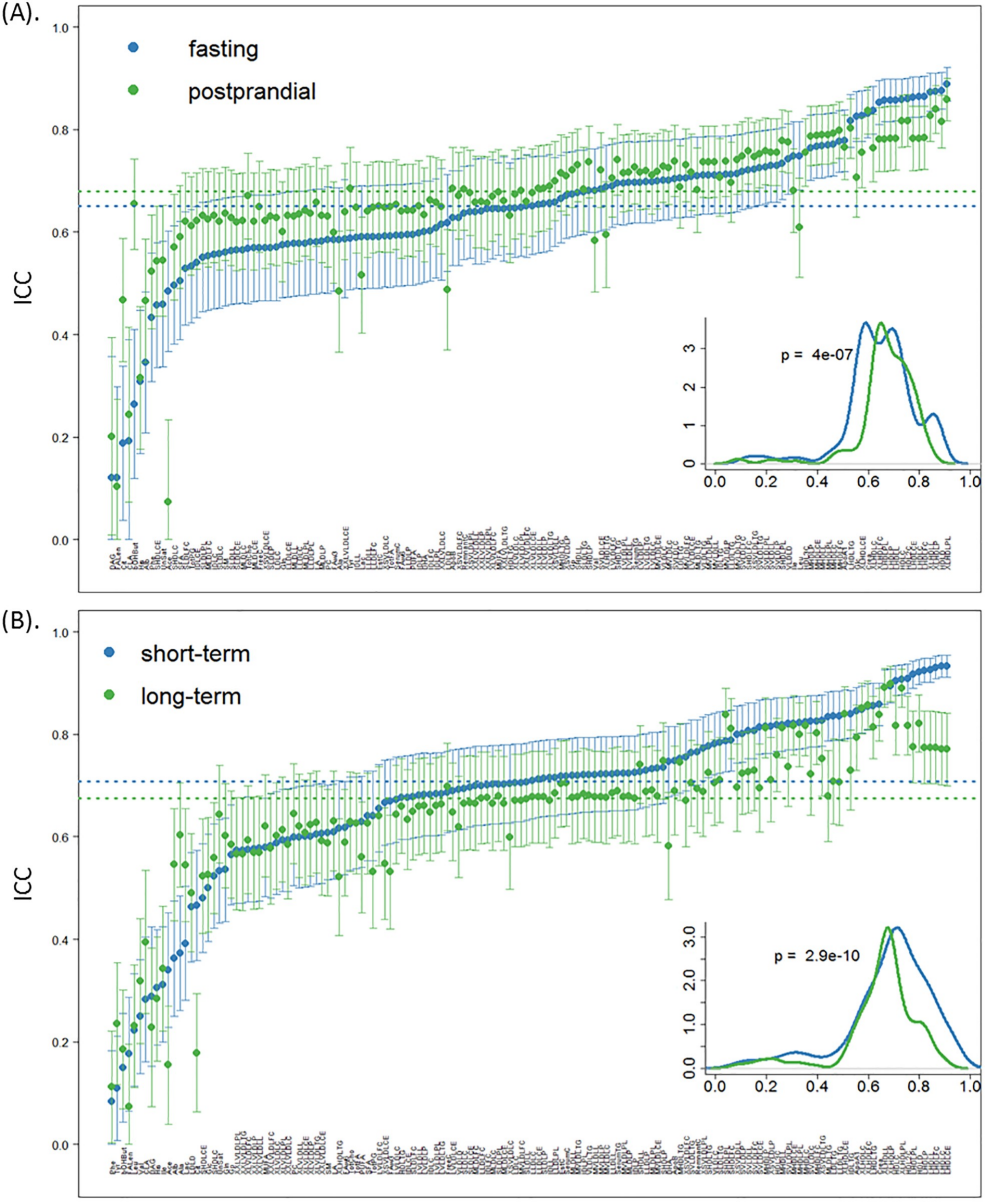
The ICC score distributions on biological reproducibility by (A) comparisons between fasting and postprandial, and (B) short- and long-term visits. Metabolites are ranked ascendingly by fasting (top) and short-term ICC scores (bottom). The lower corner represents the density plot of ICC score distributions from fasting and postprandial samples (top) or short- and long-term samples (bottom) separately, with the p-value derived from Wilcoxon two-sided test. The dash lines correspond to the median ICC scores among fasting (blue) and postprandial (green) samples (top) or short- (blue) and long-term (green) samples (bottom).

Combining the short- and long-term validation samples, fasting and postprandial states yielded median ICC scores of 0.65 and 0.68, respectively. However, as seen in Fig 2(A), the postprandial state, on average, showed less temporal biological variability (p-value: 4.0×10^−7^, 95% CI: -0.037–-0.022). Notably, 3-hydroxybutyrate revealed markedly higher ICC scores in the postprandial state (ICC: 0.26 versus 0.65) (S2 Table), and similarly, citrate and albumin also showed increased reproducibility from poor to fair (ICC: 0.19 versus 0.47 for citrate, 0.35 versus 0.47 for albumin) (S2 Table). On the other hand, several metabolite concentrations, including acetate, leucine, mean diameter of LDL particles, glucose and alanine, exhibited better reproducibility in the fasting state as compared with the postprandial state.

### Temporal biological reproducibility

To evaluate whether biological reproducibility in metabolite concentrations decreased with increasing time intervals between the measures, we determined the average ICC score for short- and long-term visits irrespective of the fasting/postprandial states (Fig 2(B)). Across all metabolite concentrations, there are on average higher ICC scores among short-term replicates than long-term replicates (Wilcoxon test p-value: 2.9×10^−10^, 95% CI of location shift of medians: 0.023–0.041). Based on the four categorical cut-off ICC scores defined in the Methods (excellent to poor reproducibility), 26 metabolite concentrations changed a category between short- and long-term repeat measures, and among these, 20 metabolite concentrations showing excellent reproducibility in the short-term (with ICC≥0.75) decreased to good in the long-term (ICC between 0.51 and 0.74). For the remaining metabolite concentrations with good reproducibility in the long-term, alanine, albumin and lactate displayed merely poor reproducibility in the short-term (short- versus long-term: 0.37 versus 0.60, 0.36 versus 0.55, 0.39 versus 0.54) (S2 Table).

### Reproducibility of fasting and postprandial state metabolite concentrations, interacting with the time interval between the measurements

**Fig 3:**
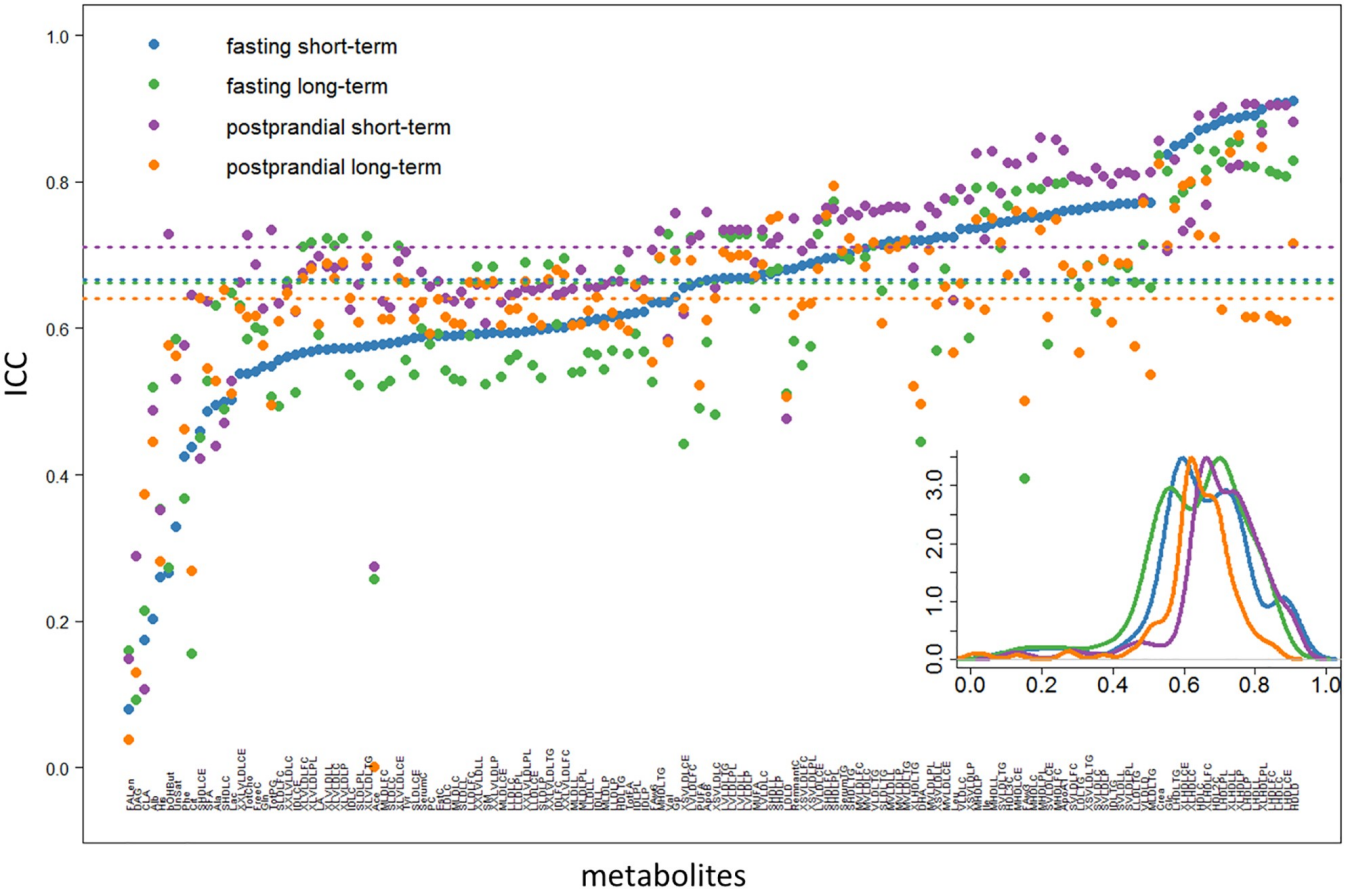
The ICC score distributions for biological reproducibility by comparisons between fasting/postprandial state, stratified on time interval between visits (short-/long-term). Metabolites are ranked ascendingly by fasting short-term (blue dots). The lower corner contains a density plot of ICC score distributions from fasting short-term (blue), fasting long-term (green), postprandial short-term (purple) and postprandial long-term (orange) separately. The dash lines correspond to the median ICC scores among four scenarios.

Subsequent to the analysis above, we revisited the question of fasting/postprandial state reproducibility conditioned on the time interval between sampling dates. When evaluating only short-term replicates, we observed an even stronger contrast between fasting and postprandial states than above with the combined full dataset (Fig 3). 3-hydroxybutyrate and the estimated degree of fatty acid unsaturation showed the largest discrepancies (ICC fasting vs. postprandial: 0.26 versus 0.73, 0.33 versus 0.53) (S2 Table). In contrast, acetate, mean diameter of LDL particles, Omega-3 fatty acids and glucose showed large biological variability in postprandial state. For long-term repeat measures solely, the ICCs for fasting and postprandial states were similar (Wilcoxon test p-value: 0.70, 95% CI of location shift of medians: -0.016–0.011) (Fig 3). Acetate and 3-hydroxybutyrate, which have been suggested for diabetes prognosis in the literature, showed large difference between fasting and postprandial states (Table 2).

**Table 2:** Technical and biological reproducibility of selected metabolic biomarkers reported in the literature.

		creatinine	albumin	citrate	acetate	3-hydroxybutyrate
Application of the biomarker		Kidney function ^(17)^	Kidney function ^(18, 19)^	Prostate cancer ^(20)^	Diabetes ^(21)^	Diabetes ^(21)^
Technical reproducibility	Fasting samples ICC (95%CI)	1.00 [1.00, 1.00]	1.00 [1.00, 1.00]	1.00 [1.00, 1.00]	1.00 [1.00, 1.00]	1.00 [1.00, 1.00]
	Postprandial samples ICC (95%CI)	1.00 [1.00, 1.00]	1.00 [1.00, 1.00]	1.00 [1.00, 1.00]	1.00 [1.00, 1.00]	1.00 [1.00, 1.00]
Short-term	Fasting ICC (95%CI)	0.82 [0.76, 0.89]	0.20 [0*, 0.42]	0.44 [0.26, 0.61]	0.58 [0.43, 0.72]	0.26 [0.060, 0.47]
	Postprandial ICC (95%CI)	0.86 [0.80, 0.91]	0.49 [0.33, 0.65]	0.64 [0.52, 0.77]	0.27 [0.077, 0.47]	0.73 [0.63, 0.83]
Long-term	Fasting ICC (95%CI)	0.84 [0.77, 0.90]	0.52 [0.36, 0.68]	0.15 [0*, 0.37]	0.26 [0.048, 0.47]	0.27 [0.067, 0.48]
	Postprandial ICC (95%CI)	0.82 [0.75, 0.90]	0.45 [0.26, 0.63]	0.27 [0.058, 0.48]	0* [0*, 0.17]	0.58 [0.43, 0.73]
Unexplained intra-individual variability (%)		15%	55%	69%	72%	70%

*minus values are round to zero.

### Multifactorial linear mixed modelling to decompose variance

**Fig 4:**
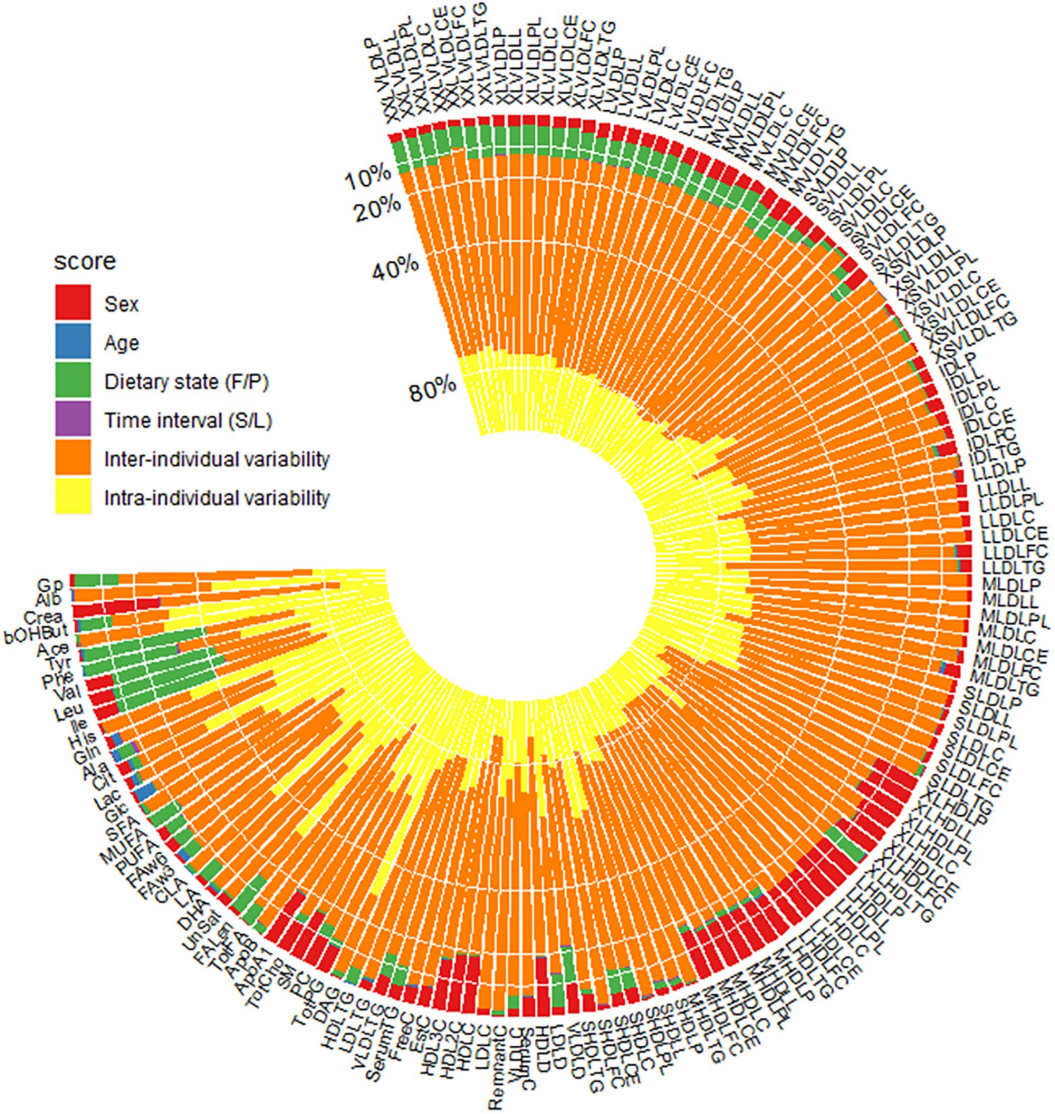
Decomposition of variance for each metabolite. Metabolites are ordered by commercial clusters. Fasting/postprandial state (F/P) corresponds to fasting (F) and postprandial; Time interval (S/L) stands for short- (S) and long-term.

To estimate the proportion of variance explained by each of the factors discussed above as well as for sex and age, we fitted each metabolite level to a multifactorial linear mixed model. The total variance of each metabolite level was decomposed into fasting/postprandial state variation, time interval between visits, sex, age, inter-individual variability and remaining intra-individual variability (this includes technical variability as well as unknown factors) (Fig 4). On average, sex and fasting/postprandial states explained 5.2% and 4.4% of the total variance, respectively. In contrast, age and time interval between visits explained less than 0.1% of the total variance. Around 66% unexplained inter-individual variability remained, however, the range was very broad across different metabolite concentrations (18% to 81%). Finally, intra-individual variability explained by biological replicates explained on average 27%. Individual metabolite concentrations were specifically influenced by different independent variables. For example, the metabolite level with the most variation explained by sex was creatinine, with 27% of the total variation explained. Overall, the effect of age on metabolite concentrations was weak, though it does appear to have an appreciable association with glucose levels, explaining 6% of the total variation. The variability of some amino acids (e.g., tyrosine, phenylalanine, valine, and leucine) was substantially attributable to the fasting/postprandial states, which suggested that these metabolite concentrations were strongly affected by the liquid meal. The metabolite concentrations with high intra-individual variations (more than 60%), included diacylglycerol, estimated description of fatty acid chain length, conjugated linoleic acid, citrate, histidine, acetate and 3-hydroxybutyrate, indicating large stochastic variations from the same individuals across measurements and less reliability for clinical prognostic and diagnostic applications (Table 2).

## Discussion

Excellent technical reproducibility of both fasting and postprandial metabolite concentrations was observed in the current study, indicating limited variation being introduced by laboratory procedures or the technology. Overall, biological reproducibility at both fasting and postprandial states and in the dichotomized short-term and long-term repeatedly measured sub-samples, was good represented by high ICC scores. Interestingly, the liquid meal challenge appeared to reduce the variability among replicates relative to their fasting state. However, this effect is attenuated with increased time interval between the measures.

### Implications for metabolic biomarkers

Creatinine levels in serum are commonly used to assess kidney function [17]. From the current study, we found that the measures of creatinine are stable both after short- and long-term measurement intervals, with equally high reproducibility in fasting and postprandial states. However, serum albumin levels as another marker for kidney function [18, 19] showed a large difference in reproducibility between fasting and postprandial states, and between short- and long-term measurement intervals. The liquid meal substantially increased the reproducibility of measures of albumin in the short-term measurement interval, while the stability of fasting albumin measures did not further decay in the long-term measurement interval. Citrate, which has been suggested as biomarker for prostate cancer [20], revealed substantial within-subject variability (~70%), which questions the utility of citrate as a stable clinical biomarker. A similar poor reproducibility was observed in acetate and 3-hydroxybutyrate from ketone bodies category, with 72% and 70% variance attributable to within-subject variability separately, which has been proposed to monitor and treat diabetes [21]. Interestingly, the liquid meal strengthened the reproducibility of 3-hydroxybutyrate, however, it led to a worse reproducibility for acetate. For 3-hydroxybutyrate, vary fasting length among participants may explain the larger variability (lower ICC scores) in the fasting state than postprandial state. For the metabolites that are outperformed (with higher ICC scores) in the fasting state, many of the compounds are likely part of the “meal” and thus variations in rates of absorption and disposal will result in increased variability in the postprandial states.

### The standardization role from a liquid meal

Previous studies have shown that both acute (24 hours prior to the visit) and prolonged (two weeks prior to the visit) standardized diet normalized fasting metabolite level measurements using ^1^H NMR spectroscopy in different types of samples. For plasma metabolite concentrations, a dietary standardization of 24 hours led to very stable inter-individual fasting metabolite concentrations, which did not substantially improve with prolonged diet standardization [22]. The observations of the current study expand upon these previous studies. Specifically, the effect of a liquid diet (150 minutes before the blood draw), reduces intra-individual variability among repeat measurements across metabolite concentrations. In principle, overnight fast is a standardization procedure to control the inter- and intra-individual variability, but it is prone to compliance issues. From the current study, we observed even stronger standardization from the liquid meal, which illustrates the potential of food challenge postprandial metabolite profiling in biomarker research. Compared with an overnight fast, a liquid meal may improve compliance to a standardization protocol and reduce the impact of inter-individual dietary choices such as previous diet intake, supplement use, and nutritional status (iron, zinc, copper, magnesium sufficiency), all of which may influence cholesterol and fatty acid levels [23].

On average, the fasting/postprandial state explained less than 2% of the total variance. Yet, for some of the specific metabolites, particularly amino acids, such as phenylalanine, valine, tyrosine and leucine, more than 30% of the variation was attributable to the fasting/postprandial state. In addition, XXL-, XL- and L-VLDL metabolite concentrations, estimated description of fatty acid chain length and monounsaturated fatty acids had ~10% of their total variation explained by fasting/postprandial state. However, the effect from the liquid meal seems greatly weakened after a repeat measure more than three years later, which might be explained by the individual characteristics changes across time far more significant and the metabolome standardization role from a liquid meal is diluted.

### The strengths and limitations

There are a number of strengths in the current study. First, the relatively large sample size allowed for the stratification biological reproducibility after short- and long-term intervals. Second, a liquid meal provided insight in the effects of transient diet standardization on reproducibility of metabolite measurements. Several limitations should also be considered. First, the study population is composed of middle-aged individuals, which might have lifestyles different from a younger population, which may have affected the fasting metabolite measurements. Second, BMI was markedly lower in the short-term reproducibility study than the long-term reproducibility, though it is unlikely that this would affect our results. Thirdly, although we observed nearly perfect technical reproducibility in the current study, the sample run scheme was not specifically designed for testing technical variability (such as batch effect). Thus, potential confounding by factors influencing sample collection, preparation, and chemical analysis cannot be excluded.

### Conclusion

Postprandial metabolite concentrations after a liquid meal measured using ^1^H NMR spectroscopy showed on average better biological reproducibility than fasting ones after a short-term repeated measure and as good as fasting metabolite concentrations after a long-term repeated measure, which indicates the robustness of applying postprandial metabolites measured after a liquid mixed meal to biomarker research.

## Supporting information

S1 Table List of measured metabolites on the platform, clustered into eleven subclasses. (DOCX)

S2 Table ICC score summary from technical and biological reproducibility analyses. (DOCX)

S1 Dataset Datasets used for the analyses. (ZIP)
