# Supplementary material for: Assessment of reproducibility and biological variability of fasting and postprandial plasma metabolite concentrations using 1H NMR spectroscopy
Source: PLoS One. 2019 Jun 20;14(6):e0218549. doi: 10.1371/journal.pone.0218549 (PMC6586348; doi:10.1371/journal.pone.0218549)
Supplement: S1 Table — (DOCX) [file pone.0218549.s001.docx]

### Supplemental material

### S1 Table. List of measured metabolites on the platform, clustered into eleven subclasses.

| **Short name** | | | **Full name** | **Remark** |
| --- | --- | --- | --- | --- |
| **Lipoprotein subclasses** | | | | |
| **Chylomicrons and extremely large VLDL particles: with an average particle diameter over 75 nm** | | | | |
| XXLVLDLP | | Total lipids (mmol/l) | |  |
| XXLVLDLL | | Phospholipids (mmol/l) | |  |
| XXLVLDLPL | | Total cholesterol (mmol/l) | |  |
| XXLVLDLC | | Cholesterol esters (mmol/l) | |  |
| XXLVLDLCE | | Free cholesterol (mmol/l) | |  |
| XXLVLDLFC | | Triglycerides (mmol/l) | |  |
| XXLVLDLTG | | Total lipids (mmol/l) | |  |
| **Very large VLDL particles: with an average particle diameter of 64 nm** | | | | |
| XLVLDLP | | Total lipids (mmol/l) | |  |
| XLVLDLL | | Phospholipids (mmol/l) | |  |
| XLVLDLPL | | Total cholesterol (mmol/l) | |  |
| XLVLDLC | | Cholesterol esters (mmol/l) | |  |
| XLVLDLCE | | Free cholesterol (mmol/l) | |  |
| XLVLDLFC | | Triglycerides (mmol/l) | |  |
| XLVLDLTG | | Total lipids (mmol/l) | |  |
| **Large VLDL particles: with an average particle diameter of 53.6 nm** | | | | |
| LVLDLP | | Total lipids (mmol/l) | |  |
| LVLDLL | | Phospholipids (mmol/l) | |  |
| LVLDLPL | | Total cholesterol (mmol/l) | |  |
| LVLDLC | | Cholesterol esters (mmol/l) | |  |
| LVLDLCE | | Free cholesterol (mmol/l) | |  |
| LVLDLFC | | Triglycerides (mmol/l) | |  |
| LVLDLTG | | Total lipids (mmol/l) | |  |
| **Medium VLDL particles: with an average particle diameter of 44.5 nm** | | | | |
| MVLDLP | | Total lipids (mmol/l) | |  |
| MVLDLL | | Phospholipids (mmol/l) | |  |
| MVLDLPL | | Total cholesterol (mmol/l) | |  |
| MVLDLC | | Cholesterol esters (mmol/l) | |  |
| MVLDLCE | | Free cholesterol (mmol/l) | |  |
| MVLDLFC | | Triglycerides (mmol/l) | |  |
| MVLDLTG | | Total lipids (mmol/l) | |  |
| **Small VLDL particles: with an average particle diameter of 36.8 nm** | | | | |
| SVLDLP | | Total lipids (mmol/l) | |  |
| SVLDLL | | Phospholipids (mmol/l) | |  |
| SVLDLPL | | Total cholesterol (mmol/l) | |  |
| SVLDLC | | Cholesterol esters (mmol/l) | |  |
| SVLDLCE | | Free cholesterol (mmol/l) | |  |
| SVLDLFC | | Triglycerides (mmol/l) | |  |
| SVLDLTG | | Total lipids (mmol/l) | |  |
| **Very small VLDL particles: with an average particle diameter of 31.3 nm** | | | | |
| XSVLDLP | | Total lipids (mmol/l) | |  |
| XSVLDLL | | Phospholipids (mmol/l) | |  |
| XSVLDLPL | | Total cholesterol (mmol/l) | |  |
| XSVLDLC | | Cholesterol esters (mmol/l) | |  |
| XSVLDLCE | | Free cholesterol (mmol/l) | |  |
| XSVLDLFC | | Triglycerides (mmol/l) | |  |
| XSVLDLTG | | Total lipids (mmol/l) | |  |
| **IDL particles: intermediate-density lipoprotein particles with an average particle diameter of 28.6 nm** | | | | |
| IDLP | | Total lipids (mmol/l) | |  |
| IDLL | | Phospholipids (mmol/l) | |  |
| IDLPL | | Total cholesterol (mmol/l) | |  |
| IDLC | | Cholesterol esters (mmol/l) | |  |
| IDLCE | | Free cholesterol (mmol/l) | |  |
| IDLFC | | Triglycerides (mmol/l) | |  |
| IDLTG | | Total lipids (mmol/l) | |  |
| **Large LDL particles: low-density lipoprotein particles with an average particle diameter of 25.5 nm** | | | | |
| LLDLP | | Total lipids (mmol/l) | |  |
| LLDLL | | Phospholipids (mmol/l) | |  |
| LLDLPL | | Total cholesterol (mmol/l) | |  |
| LLDLC | | Cholesterol esters (mmol/l) | |  |
| LLDLCE | | Free cholesterol (mmol/l) | |  |
| LLDLFC | | Triglycerides (mmol/l) | |  |
| LLDLTG | | Total lipids (mmol/l) | |  |
| **Medium LDL particles: low-density lipoprotein particles with an average particle diameter of 23.0 nm** | | | | |
| MLDLP | | Total lipids (mmol/l) | |  |
| MLDLL | | Phospholipids (mmol/l) | |  |
| MLDLPL | | Total cholesterol (mmol/l) | |  |
| MLDLC | | Cholesterol esters (mmol/l) | |  |
| MLDLCE | | Free cholesterol (mmol/l) | |  |
| MLDLFC | | Triglycerides (mmol/l) | |  |
| MLDLTG | | Total lipids (mmol/l) | |  |
| **Small LDL particles: low-density lipoprotein particles with an average particle diameter of 18.7 nm** | | | | |
| SLDLP | | Total lipids (mmol/l) | |  |
| SLDLL | | Phospholipids (mmol/l) | |  |
| SLDLPL | | Total cholesterol (mmol/l) | |  |
| SLDLC | | Cholesterol esters (mmol/l) | |  |
| SLDLCE | | Free cholesterol (mmol/l) | |  |
| SLDLFC | | Triglycerides (mmol/l) | |  |
| SLDLTG | | Total lipids (mmol/l) | |  |
| **Very large HDL particles: high-density lipoprotein particles with an average particle diameter of 14.3 nm** | | | | |
| XLHDLP | | Total lipids (mmol/l) | |  |
| XLHDLL | | Phospholipids (mmol/l) | |  |
| XLHDLPL | | Total cholesterol (mmol/l) | |  |
| XLHDLC | | Cholesterol esters (mmol/l) | |  |
| XLHDLCE | | Free cholesterol (mmol/l) | |  |
| XLHDLFC | | Triglycerides (mmol/l) | |  |
| XLHDLTG | | Total lipids (mmol/l) | |  |
| **Large HDL particles: high-density lipoprotein particles with an average particle diameter of 12.1 nm** | | | | |
| LHDLP | Total lipids (mmol/l) | | |  |
| LHDLL | Phospholipids (mmol/l) | | |  |
| LHDLPL | Total cholesterol (mmol/l) | | |  |
| LHDLC | Cholesterol esters (mmol/l) | | |  |
| LHDLCE | Free cholesterol (mmol/l) | | |  |
| LHDLFC | Triglycerides (mmol/l) | | |  |
| LHDLTG | Total lipids (mmol/l) | | |  |
| **Medium HDL particles: high-density lipoprotein particles with an average particle diameter of 10.9 nm** | | | | |
| MHDLP | Total lipids (mmol/l) | | |  |
| MHDLL | Phospholipids (mmol/l) | | |  |
| MHDLPL | Total cholesterol (mmol/l) | | |  |
| MHDLC | Cholesterol esters (mmol/l) | | |  |
| MHDLCE | Free cholesterol (mmol/l) | | |  |
| MHDLFC | Triglycerides (mmol/l) | | |  |
| MHDLTG | Total lipids (mmol/l) | | |  |
| **Small HDL particles** | | | | |
| SHDLP | Total lipids (mmol/l) | | |  |
| SHDLL | Phospholipids (mmol/l) | | |  |
| SHDLPL | Total cholesterol (mmol/l) | | |  |
| SHDLC | Cholesterol esters (mmol/l) | | |  |
| SHDLCE | Free cholesterol (mmol/l) | | |  |
| SHDLFC | Triglycerides (mmol/l) | | |  |
| SHDLTG | Total lipids (mmol/l) | | |  |
| **Lipoprotein particle sizes** | | | | |
| VLDLD | Mean diameter of VLDL particles (nm) | | | calculated as the particle concentration weighted average of the XXL-, XL-, L-, M-, S, and XS-VLDL subclass diameters |
| LDLD | Mean diameter of LDL particles (nm) | | | calculated as the particle concentration weighted average of all the LDL and the IDL subclass diameters |
| HDLD | Mean diameter of HDL particles (nm) | | | calculated as the particle concentration weighted average of all the HDL subclass diameters |
| **Cholesterol** | | | | |
| SerumC | Serum total cholesterol (mmol/l) | | |  |
| VLDLC | Total cholesterol in VLDL(mmol/l) | | |  |
| RemnantC | Remnant cholesterol (non-HDL, non-LDL-cholesterol) (mmol/l) | | |  |
| LDLC | Total cholesterol in LDL (mmol/l) | | |  |
| HDLC | Total cholesterol in HDL (mmol/l) | | |  |
| HDL2C | Total cholesterol in HDL2 (mmol/l) | | | HDL particles within the density range of 1.063-1.125 g/mL |
| HDL3C | Total cholesterol in HDL3 (mmol/l) | | | HDL particles within the density range of 1.125-1.210 g/mL |
| EstC | Esterified cholesterol (mmol/l) | | |  |
| FreeC | Free cholesterol (mmol/l) | | |  |
| **Glycerides & phospholipids** | | | | |
| SerumTG | Serum total triglycerides (mmol/l) | | |  |
| VLDLTG | Triglycerides in VLDL (mmol/l) | | |  |
| LDLTG | Triglycerides in LDL (mmol/l) | | |  |
| HDLTG | Triglycerides in HDL(mmol/l) | | |  |
| DAG | Diacylglycerol (mmol/l) | | |  |
| TotPG | Total phosphoglycerides (mmol/l) | | |  |
| PC | Phosphatidylcholine and other cholines (mmol/l) | | |  |
| SM | Sphingomyelins (mmol/l) | | |  |
| TotCho | Total cholines (mmol/l) | | |  |
| **Apolipoproteins** | | | | |
| ApoA1 | Apolipoprotein A-I (g/l) | | |  |
| ApoB | Apolipoprotein B (g/l) | | |  |
| **Fatty acids (FA) & saturation** | | | | |
| TotFA | Total fatty acids (mmol/l) | | |  |
| FALen | Estimated description of fatty acid chain length, not actual carbon number | | |  |
| UnSat | Estimated degree of unsaturation | | |  |
| DHA | 22:6, docosahexaenoic acid (mmol/l) | | |  |
| LA | 18:2, linoleic acid (mmol/l) | | |  |
| CLA | Conjugated linoleic acid - mmol/l | | |  |
| FAw3 | Omega-3 fatty acids (mmol/l) | | |  |
| FAw6 | Omega-6 fatty acids (mmol/l) | | |  |
| PUFA | Polyunsaturated fatty acids (mmol/l) | | |  |
| MUFA | Monounsaturated fatty acids; 16:1, 18:1 (mmol/l) | | |  |
| SFA | Saturated fatty acids (mmol/l) | | |  |
| **Glycolysis related metabolites** | | | | |
| Glc | Glucose (mmol/l) | | |  |
| Lac | Lactate (mmol/l) | | |  |
| Cit | Citrate (mmol/l) | | |  |
| **Amino acids** | | | | |
| Ala | Alanine (mmol/l) | | |  |
| Gln | Glutamine (mmol/l) | | |  |
| His | Histidine (mmol/l) | | |  |
| Ile | Isoleucine (mmol/l) | | |  |
| Leu | Leucine (mmol/l) | | |  |
| Val | Valine (mmol/l) | | |  |
| Phe | Phenylalanine (mmol/l) | | |  |
| Tyr | Tyrosine (mmol/l) | | |  |
| **Ketone bodies** | | | | |
| Ace | Acetate (mmol/l) | | |  |
| bOHBut | 3-hydroxybutyrate (mmol/l) | | |  |
| **Fluid balance** | | | | |
| Crea | Creatinine (mmol/l) | | |  |
| Alb | Albumin | | |  |
| **Inflammation** | | | | |
| Gp | Glycoprotein acetyls, mainly a1-acid glycoprotein (mmol/l) | | |  |
