# Supplementary material for: Assessment of reproducibility and biological variability of fasting and postprandial plasma metabolite concentrations using 1H NMR spectroscopy
Source: PLoS One. 2019 Jun 20;14(6):e0218549. doi: 10.1371/journal.pone.0218549 (PMC6586348; doi:10.1371/journal.pone.0218549)
Supplement: S2 Table — (DOCX) [file pone.0218549.s002.docx]

### Supplemental material

### S2 Table. ICC score summary from technical and biological reproducibility analyses.

|  | fasting-state | postprandial-state | short-term | long-term | fasting-short-term | fasting-long-term | postprandial-short-term | postprandial-long-term | technical-fasting | technical-postprandial |
| --- | --- | --- | --- | --- | --- | --- | --- | --- | --- | --- |
| XXLVLDLP | 0.64 | 0.69 | 0.59 | 0.59 | 0.60 | 0.69 | 0.70 | 0.67 | 1.00 | 1.00 |
| XXLVLDLL | 0.65 | 0.69 | 0.60 | 0.59 | 0.61 | 0.69 | 0.71 | 0.67 | 1.00 | 1.00 |
| XXLVLDLPL | 0.64 | 0.68 | 0.59 | 0.58 | 0.59 | 0.69 | 0.70 | 0.67 | 1.00 | 1.00 |
| XXLVLDLC | 0.67 | 0.68 | 0.63 | 0.61 | 0.64 | 0.69 | 0.71 | 0.66 | 1.00 | 1.00 |
| XXLVLDLCE | 0.68 | 0.67 | 0.65 | 0.62 | 0.69 | 0.68 | 0.72 | 0.64 | 1.00 | 1.00 |
| XXLVLDLFC | 0.65 | 0.69 | 0.59 | 0.59 | 0.60 | 0.70 | 0.69 | 0.68 | 1.00 | 1.00 |
| XXLVLDLTG | 0.64 | 0.69 | 0.59 | 0.58 | 0.59 | 0.68 | 0.70 | 0.67 | 1.00 | 1.00 |
| XLVLDLP | 0.69 | 0.71 | 0.65 | 0.63 | 0.67 | 0.70 | 0.71 | 0.70 | 1.00 | 1.00 |
| XLVLDLL | 0.69 | 0.70 | 0.65 | 0.63 | 0.67 | 0.70 | 0.71 | 0.70 | 1.00 | 1.00 |
| XLVLDLPL | 0.68 | 0.70 | 0.65 | 0.62 | 0.66 | 0.70 | 0.71 | 0.69 | 1.00 | 1.00 |
| XLVLDLC | 0.68 | 0.69 | 0.64 | 0.61 | 0.67 | 0.71 | 0.71 | 0.68 | 1.00 | 1.00 |
| XLVLDLCE | 0.69 | 0.70 | 0.66 | 0.62 | 0.68 | 0.71 | 0.72 | 0.68 | 1.00 | 1.00 |
| XLVLDLFC | 0.68 | 0.69 | 0.63 | 0.61 | 0.65 | 0.70 | 0.70 | 0.68 | 1.00 | 1.00 |
| XLVLDLTG | 0.69 | 0.71 | 0.66 | 0.64 | 0.68 | 0.70 | 0.71 | 0.71 | 1.00 | 1.00 |
| LVLDLP | 0.69 | 0.72 | 0.69 | 0.66 | 0.69 | 0.71 | 0.74 | 0.71 | 1.00 | 1.00 |
| LVLDLL | 0.69 | 0.72 | 0.69 | 0.66 | 0.69 | 0.71 | 0.74 | 0.71 | 1.00 | 1.00 |
| LVLDLPL | 0.69 | 0.72 | 0.69 | 0.66 | 0.69 | 0.70 | 0.74 | 0.71 | 1.00 | 1.00 |
| LVLDLC | 0.70 | 0.72 | 0.68 | 0.65 | 0.69 | 0.71 | 0.74 | 0.70 | 1.00 | 1.00 |
| LVLDLCE | 0.71 | 0.72 | 0.70 | 0.66 | 0.71 | 0.72 | 0.75 | 0.69 | 1.00 | 1.00 |
| LVLDLFC | 0.69 | 0.71 | 0.66 | 0.64 | 0.67 | 0.71 | 0.72 | 0.70 | 1.00 | 1.00 |
| LVLDLTG | 0.69 | 0.73 | 0.69 | 0.67 | 0.69 | 0.70 | 0.74 | 0.72 | 1.00 | 1.00 |
| MVLDLP | 0.71 | 0.74 | 0.72 | 0.68 | 0.72 | 0.71 | 0.77 | 0.71 | 1.00 | 1.00 |
| MVLDLL | 0.71 | 0.74 | 0.72 | 0.68 | 0.72 | 0.71 | 0.77 | 0.71 | 1.00 | 1.00 |
| MVLDLPL | 0.71 | 0.74 | 0.72 | 0.68 | 0.72 | 0.71 | 0.77 | 0.71 | 1.00 | 1.00 |
| MVLDLC | 0.70 | 0.73 | 0.72 | 0.68 | 0.71 | 0.70 | 0.77 | 0.68 | 1.00 | 1.00 |
| MVLDLCE | 0.70 | 0.72 | 0.74 | 0.68 | 0.71 | 0.68 | 0.78 | 0.66 | 1.00 | 1.00 |
| MVLDLFC | 0.71 | 0.73 | 0.71 | 0.67 | 0.71 | 0.71 | 0.75 | 0.71 | 1.00 | 1.00 |
| MVLDLTG | 0.71 | 0.74 | 0.72 | 0.68 | 0.72 | 0.71 | 0.76 | 0.72 | 1.00 | 1.00 |
| SVLDLP | 0.73 | 0.76 | 0.80 | 0.72 | 0.77 | 0.69 | 0.81 | 0.69 | 1.00 | 1.00 |
| SVLDLL | 0.73 | 0.76 | 0.81 | 0.73 | 0.77 | 0.69 | 0.81 | 0.69 | 1.00 | 1.00 |
| SVLDLPL | 0.73 | 0.76 | 0.82 | 0.74 | 0.77 | 0.68 | 0.81 | 0.69 | 1.00 | 1.00 |
| SVLDLC | 0.70 | 0.74 | 0.83 | 0.72 | 0.77 | 0.62 | 0.82 | 0.63 | 1.00 | 1.00 |
| SVLDLCE | 0.68 | 0.72 | 0.81 | 0.70 | 0.75 | 0.58 | 0.80 | 0.62 | 1.00 | 1.00 |
| SVLDLFC | 0.72 | 0.75 | 0.81 | 0.73 | 0.76 | 0.67 | 0.81 | 0.68 | 1.00 | 1.00 |
| SVLDLTG | 0.72 | 0.75 | 0.76 | 0.71 | 0.74 | 0.71 | 0.78 | 0.72 | 1.00 | 1.00 |
| XSVLDLP | 0.67 | 0.71 | 0.82 | 0.71 | 0.74 | 0.59 | 0.78 | 0.63 | 1.00 | 1.00 |
| XSVLDLL | 0.66 | 0.70 | 0.80 | 0.70 | 0.72 | 0.57 | 0.76 | 0.63 | 1.00 | 1.00 |
| XSVLDLPL | 0.64 | 0.68 | 0.77 | 0.69 | 0.69 | 0.58 | 0.72 | 0.63 | 1.00 | 1.00 |
| XSVLDLC | 0.59 | 0.65 | 0.70 | 0.60 | 0.67 | 0.48 | 0.65 | 0.64 | 1.00 | 1.00 |
| XSVLDLCE | 0.57 | 0.62 | 0.67 | 0.55 | 0.66 | 0.44 | 0.62 | 0.63 | 1.00 | 1.00 |
| XSVLDLFC | 0.63 | 0.67 | 0.76 | 0.67 | 0.69 | 0.55 | 0.71 | 0.63 | 1.00 | 1.00 |
| XSVLDLTG | 0.72 | 0.75 | 0.83 | 0.75 | 0.76 | 0.68 | 0.80 | 0.68 | 1.00 | 0.97 |
| IDLP | 0.60 | 0.65 | 0.73 | 0.68 | 0.62 | 0.57 | 0.67 | 0.64 | 1.00 | 1.00 |
| IDLL | 0.59 | 0.65 | 0.72 | 0.67 | 0.61 | 0.57 | 0.65 | 0.64 | 1.00 | 1.00 |
| IDLPL | 0.61 | 0.66 | 0.72 | 0.68 | 0.62 | 0.59 | 0.66 | 0.66 | 1.00 | 1.00 |
| IDLC | 0.56 | 0.63 | 0.68 | 0.65 | 0.57 | 0.54 | 0.63 | 0.64 | 1.00 | 1.00 |
| IDLCE | 0.54 | 0.62 | 0.68 | 0.63 | 0.57 | 0.51 | 0.62 | 0.62 | 1.00 | 1.00 |
| IDLFC | 0.60 | 0.66 | 0.70 | 0.68 | 0.60 | 0.60 | 0.65 | 0.68 | 1.00 | 1.00 |
| IDLTG | 0.71 | 0.71 | 0.84 | 0.73 | 0.77 | 0.66 | 0.80 | 0.61 | 1.00 | 1.00 |
| LLDLP | 0.60 | 0.64 | 0.73 | 0.69 | 0.62 | 0.57 | 0.66 | 0.62 | 1.00 | 1.00 |
| LLDLL | 0.59 | 0.64 | 0.72 | 0.69 | 0.61 | 0.57 | 0.66 | 0.62 | 1.00 | 1.00 |
| LLDLPL | 0.58 | 0.64 | 0.72 | 0.69 | 0.59 | 0.57 | 0.65 | 0.63 | 1.00 | 1.00 |
| LLDLC | 0.58 | 0.64 | 0.71 | 0.68 | 0.60 | 0.56 | 0.65 | 0.62 | 1.00 | 1.00 |
| LLDLCE | 0.58 | 0.63 | 0.71 | 0.68 | 0.60 | 0.55 | 0.65 | 0.61 | 1.00 | 1.00 |
| LLDLFC | 0.59 | 0.65 | 0.70 | 0.68 | 0.59 | 0.59 | 0.63 | 0.66 | 1.00 | 1.00 |
| LLDLTG | 0.71 | 0.70 | 0.84 | 0.71 | 0.77 | 0.66 | 0.81 | 0.58 | 1.00 | 1.00 |
| MLDLP | 0.58 | 0.62 | 0.72 | 0.67 | 0.61 | 0.55 | 0.66 | 0.59 | 1.00 | 1.00 |
| MLDLL | 0.58 | 0.62 | 0.72 | 0.67 | 0.61 | 0.54 | 0.65 | 0.59 | 1.00 | 1.00 |
| MLDLPL | 0.58 | 0.64 | 0.73 | 0.69 | 0.61 | 0.54 | 0.68 | 0.61 | 1.00 | 1.00 |
| MLDLC | 0.57 | 0.62 | 0.70 | 0.66 | 0.59 | 0.53 | 0.64 | 0.60 | 1.00 | 1.00 |
| MLDLCE | 0.57 | 0.62 | 0.70 | 0.67 | 0.60 | 0.53 | 0.64 | 0.60 | 1.00 | 1.00 |
| MLDLFC | 0.55 | 0.63 | 0.70 | 0.67 | 0.58 | 0.52 | 0.64 | 0.62 | 1.00 | 1.00 |
| MLDLTG | 0.71 | 0.68 | 0.83 | 0.68 | 0.77 | 0.66 | 0.81 | 0.54 | 1.00 | 1.00 |
| SLDLP | 0.57 | 0.62 | 0.71 | 0.67 | 0.60 | 0.53 | 0.66 | 0.58 | 1.00 | 1.00 |
| SLDLL | 0.56 | 0.61 | 0.71 | 0.66 | 0.59 | 0.53 | 0.65 | 0.58 | 1.00 | 1.00 |
| SLDLPL | 0.55 | 0.63 | 0.70 | 0.68 | 0.57 | 0.52 | 0.66 | 0.61 | 1.00 | 1.00 |
| SLDLC | 0.56 | 0.62 | 0.69 | 0.66 | 0.58 | 0.53 | 0.63 | 0.60 | 1.00 | 1.00 |
| SLDLCE | 0.57 | 0.62 | 0.70 | 0.67 | 0.59 | 0.54 | 0.63 | 0.61 | 1.00 | 1.00 |
| SLDLFC | 0.53 | 0.62 | 0.68 | 0.65 | 0.56 | 0.50 | 0.64 | 0.61 | 1.00 | 1.00 |
| SLDLTG | 0.68 | 0.65 | 0.78 | 0.69 | 0.71 | 0.65 | 0.76 | 0.54 | 1.00 | 1.00 |
| XLHDLP | 0.86 | 0.84 | 0.89 | 0.88 | 0.88 | 0.82 | 0.84 | 0.83 | 1.00 | 1.00 |
| XLHDLL | 0.86 | 0.84 | 0.89 | 0.88 | 0.88 | 0.82 | 0.84 | 0.83 | 1.00 | 1.00 |
| XLHDLPL | 0.87 | 0.85 | 0.91 | 0.87 | 0.89 | 0.84 | 0.87 | 0.82 | 1.00 | 1.00 |
| XLHDLC | 0.83 | 0.78 | 0.85 | 0.84 | 0.86 | 0.78 | 0.77 | 0.81 | 1.00 | 1.00 |
| XLHDLCE | 0.82 | 0.78 | 0.84 | 0.83 | 0.85 | 0.77 | 0.77 | 0.81 | 1.00 | 1.00 |
| XLHDLFC | 0.85 | 0.79 | 0.86 | 0.85 | 0.88 | 0.81 | 0.80 | 0.77 | 1.00 | 1.00 |
| XLHDLTG | 0.68 | 0.63 | 0.65 | 0.50 | 0.72 | 0.64 | 0.74 | 0.50 | 1.00 | 1.00 |
| LHDLP | 0.87 | 0.82 | 0.92 | 0.80 | 0.90 | 0.83 | 0.90 | 0.70 | 1.00 | 1.00 |
| LHDLL | 0.87 | 0.82 | 0.92 | 0.80 | 0.90 | 0.83 | 0.90 | 0.70 | 1.00 | 1.00 |
| LHDLPL | 0.86 | 0.82 | 0.90 | 0.80 | 0.88 | 0.83 | 0.89 | 0.72 | 1.00 | 1.00 |
| LHDLC | 0.87 | 0.82 | 0.93 | 0.80 | 0.90 | 0.82 | 0.90 | 0.69 | 1.00 | 1.00 |
| LHDLCE | 0.87 | 0.82 | 0.93 | 0.80 | 0.91 | 0.82 | 0.90 | 0.69 | 1.00 | 1.00 |
| LHDLFC | 0.87 | 0.81 | 0.93 | 0.80 | 0.90 | 0.83 | 0.91 | 0.68 | 1.00 | 1.00 |
| LHDLTG | 0.80 | 0.82 | 0.83 | 0.78 | 0.84 | 0.73 | 0.83 | 0.81 | 1.00 | 1.00 |
| MHDLP | 0.76 | 0.79 | 0.81 | 0.81 | 0.73 | 0.79 | 0.84 | 0.75 | 1.00 | 1.00 |
| MHDLL | 0.77 | 0.79 | 0.82 | 0.81 | 0.74 | 0.79 | 0.84 | 0.75 | 1.00 | 1.00 |
| MHDLPL | 0.77 | 0.79 | 0.82 | 0.80 | 0.75 | 0.79 | 0.86 | 0.73 | 1.00 | 1.00 |
| MHDLC | 0.77 | 0.79 | 0.82 | 0.82 | 0.75 | 0.79 | 0.83 | 0.76 | 1.00 | 1.00 |
| MHDLCE | 0.77 | 0.79 | 0.82 | 0.82 | 0.75 | 0.79 | 0.82 | 0.76 | 1.00 | 1.00 |
| MHDLFC | 0.78 | 0.80 | 0.83 | 0.80 | 0.76 | 0.80 | 0.86 | 0.75 | 1.00 | 1.00 |
| MHDLTG | 0.67 | 0.72 | 0.75 | 0.75 | 0.64 | 0.70 | 0.73 | 0.70 | 1.00 | 1.00 |
| SHDLP | 0.68 | 0.74 | 0.73 | 0.75 | 0.67 | 0.68 | 0.73 | 0.75 | 1.00 | 1.00 |
| SHDLL | 0.67 | 0.73 | 0.73 | 0.74 | 0.67 | 0.68 | 0.72 | 0.75 | 1.00 | 1.00 |
| SHDLPL | 0.75 | 0.78 | 0.80 | 0.84 | 0.73 | 0.77 | 0.76 | 0.79 | 1.00 | 1.00 |
| SHDLC | 0.49 | 0.57 | 0.52 | 0.56 | 0.50 | 0.48 | 0.47 | 0.65 | 1.00 | 1.00 |
| SHDLCE | 0.46 | 0.54 | 0.48 | 0.52 | 0.46 | 0.45 | 0.42 | 0.64 | 1.00 | 1.00 |
| SHDLFC | 0.74 | 0.76 | 0.80 | 0.81 | 0.73 | 0.75 | 0.76 | 0.75 | 1.00 | 1.00 |
| SHDLTG | 0.69 | 0.74 | 0.78 | 0.73 | 0.70 | 0.69 | 0.76 | 0.72 | 1.00 | 1.00 |
| VLDLD | 0.74 | 0.77 | 0.69 | 0.62 | 0.77 | 0.71 | 0.78 | 0.77 | 1.00 | 1.00 |
| LDLD | 0.62 | 0.49 | 0.46 | 0.49 | 0.68 | 0.51 | 0.48 | 0.51 | 1.00 | 1.00 |
| HDLD | 0.87 | 0.81 | 0.92 | 0.82 | 0.91 | 0.83 | 0.88 | 0.71 | 1.00 | 1.00 |
| SerumC | 0.59 | 0.66 | 0.72 | 0.71 | 0.59 | 0.60 | 0.68 | 0.64 | 1.00 | 1.00 |
| VLDLC | 0.70 | 0.73 | 0.78 | 0.71 | 0.73 | 0.66 | 0.79 | 0.66 | 1.00 | 1.00 |
| RemnantC | 0.64 | 0.69 | 0.76 | 0.70 | 0.68 | 0.58 | 0.75 | 0.62 | 1.00 | 1.00 |
| LDLC | 0.57 | 0.63 | 0.71 | 0.67 | 0.59 | 0.54 | 0.64 | 0.62 | 1.00 | 1.00 |
| HDLC | 0.86 | 0.82 | 0.90 | 0.82 | 0.87 | 0.84 | 0.89 | 0.73 | 1.00 | 1.00 |
| HDL2C | 0.86 | 0.82 | 0.91 | 0.82 | 0.88 | 0.84 | 0.89 | 0.72 | 1.00 | 1.00 |
| HDL3C | 0.76 | 0.76 | 0.82 | 0.76 | 0.74 | 0.77 | 0.83 | 0.68 | 1.00 | 1.00 |
| EstC | 0.59 | 0.65 | 0.72 | 0.70 | 0.59 | 0.59 | 0.66 | 0.64 | 1.00 | 1.00 |
| FreeC | 0.57 | 0.65 | 0.69 | 0.70 | 0.54 | 0.60 | 0.69 | 0.62 | 0.99 | 0.99 |
| SerumTG | 0.70 | 0.73 | 0.72 | 0.69 | 0.70 | 0.70 | 0.75 | 0.71 | 1.00 | 1.00 |
| VLDLTG | 0.71 | 0.74 | 0.72 | 0.68 | 0.71 | 0.71 | 0.76 | 0.72 | 1.00 | 1.00 |
| LDLTG | 0.70 | 0.69 | 0.84 | 0.71 | 0.76 | 0.66 | 0.80 | 0.57 | 1.00 | 1.00 |
| HDLTG | 0.65 | 0.63 | 0.68 | 0.66 | 0.62 | 0.68 | 0.66 | 0.60 | 1.00 | 1.00 |
| DAG | 0.26 | 0.16 | 0.35 | 0.21 | 0.31 | 0.17 | 0.43 | -0.04 | 1.00 | 0.96 |
| TotPG | 0.53 | 0.61 | 0.64 | 0.53 | 0.55 | 0.51 | 0.73 | 0.50 | 1.00 | 0.99 |
| PC | 0.59 | 0.63 | 0.60 | 0.65 | 0.59 | 0.58 | 0.66 | 0.59 | 1.00 | 0.99 |
| SM | 0.56 | 0.64 | 0.61 | 0.59 | 0.59 | 0.53 | 0.61 | 0.66 | 1.00 | 0.98 |
| TotCho | 0.57 | 0.67 | 0.63 | 0.63 | 0.54 | 0.59 | 0.73 | 0.62 | 1.00 | 0.99 |
| ApoA1 | 0.78 | 0.77 | 0.85 | 0.79 | 0.76 | 0.80 | 0.84 | 0.69 | 1.00 | 1.00 |
| ApoB | 0.63 | 0.69 | 0.75 | 0.69 | 0.66 | 0.58 | 0.76 | 0.61 | 1.00 | 1.00 |
| TotFA | 0.59 | 0.65 | 0.63 | 0.63 | 0.62 | 0.56 | 0.70 | 0.60 | 0.99 | 0.99 |
| FALen | 0.12 | 0.10 | 0.18 | 0.07 | 0.08 | 0.16 | 0.15 | 0.04 | 0.77 | 0.83 |
| UnSat | 0.46 | 0.55 | 0.53 | 0.64 | 0.32 | 0.58 | 0.53 | 0.56 | 0.96 | 0.88 |
| DHA | 0.60 | 0.64 | 0.75 | 0.58 | 0.72 | 0.44 | 0.74 | 0.50 | 1.00 | 0.93 |
| LA | 0.58 | 0.66 | 0.61 | 0.63 | 0.57 | 0.59 | 0.70 | 0.61 | 1.00 | 0.99 |
| CLA | 0.31 | 0.30 | 0.33 | 0.43 | 0.27 | 0.37 | 0.16 | 0.43 | 1.00 | 0.97 |
| FAw3 | 0.58 | 0.60 | 0.67 | 0.53 | 0.75 | 0.39 | 0.68 | 0.50 | 1.00 | 0.95 |
| FAw6 | 0.59 | 0.64 | 0.62 | 0.59 | 0.63 | 0.53 | 0.71 | 0.56 | 1.00 | 0.98 |
| PUFA | 0.59 | 0.64 | 0.63 | 0.56 | 0.66 | 0.49 | 0.73 | 0.53 | 1.00 | 0.97 |
| MUFA | 0.64 | 0.68 | 0.58 | 0.62 | 0.67 | 0.63 | 0.69 | 0.67 | 1.00 | 1.00 |
| SFA | 0.50 | 0.59 | 0.64 | 0.62 | 0.48 | 0.53 | 0.64 | 0.54 | 0.98 | 0.98 |
| Glc | 0.83 | 0.71 | 0.50 | 0.53 | 0.84 | 0.82 | 0.71 | 0.71 | 1.00 | 1.00 |
| Lac | 0.59 | 0.52 | 0.39 | 0.55 | 0.50 | 0.65 | 0.53 | 0.51 | 1.00 | 1.00 |
| Cit | 0.19 | 0.47 | 0.47 | 0.18 | 0.44 | 0.15 | 0.64 | 0.27 | 1.00 | 1.00 |
| Ala | 0.59 | 0.48 | 0.37 | 0.60 | 0.50 | 0.63 | 0.44 | 0.53 | 1.00 | 1.00 |
| Gln | 0.58 | 0.60 | 0.54 | 0.60 | 0.55 | 0.60 | 0.63 | 0.58 | 1.00 | 1.00 |
| His | 0.31 | 0.32 | 0.31 | 0.28 | 0.26 | 0.35 | 0.35 | 0.28 | 1.00 | 1.00 |
| Ile | 0.75 | 0.68 | 0.31 | 0.34 | 0.74 | 0.76 | 0.72 | 0.62 | 1.00 | 1.00 |
| Leu | 0.75 | 0.61 | 0.22 | 0.23 | 0.72 | 0.77 | 0.64 | 0.56 | 1.00 | 1.00 |
| Val | 0.68 | 0.58 | 0.25 | 0.32 | 0.64 | 0.73 | 0.58 | 0.58 | 1.00 | 1.00 |
| Phe | 0.43 | 0.52 | 0.08 | 0.11 | 0.42 | 0.37 | 0.58 | 0.46 | 1.00 | 1.00 |
| Tyr | 0.59 | 0.68 | 0.11 | 0.23 | 0.58 | 0.56 | 0.70 | 0.66 | 1.00 | 1.00 |
| Ace | 0.49 | 0.07 | 0.34 | 0.15 | 0.58 | 0.26 | 0.27 | -0.08 | 1.00 | 1.00 |
| bOHBut | 0.26 | 0.65 | 0.15 | 0.19 | 0.26 | 0.27 | 0.73 | 0.58 | 1.00 | 1.00 |
| Crea | 0.83 | 0.84 | 0.86 | 0.84 | 0.82 | 0.83 | 0.86 | 0.83 | 1.00 | 1.00 |
| Alb | 0.35 | 0.46 | 0.36 | 0.55 | 0.20 | 0.52 | 0.49 | 0.44 | 1.00 | 1.00 |
| Gp | 0.67 | 0.72 | 0.56 | 0.58 | 0.64 | 0.71 | 0.76 | 0.69 | 1.00 | 1.00 |
